# PVCTools: parallel variation calling tools

**DOI:** 10.1016/j.heliyon.2019.e02530

**Published:** 2019-10-03

**Authors:** Jingjing Jin, Jiajun Liu, Yelin Yin, Zefeng Li, Peng Lu, Yalong Xu, Jianfeng Zhang, Peijian Cao, Dasha Hu

**Affiliations:** a China Tobacco Gene Research Center, Zhengzhou Tobacco Research Institute of CNTC, Zhengzhou, 450001, China; b Computer Science and Technology, Sichuan University, Chengdu, 450000, China; c Zhengzhou Tongbiao Environmental Testing Co., LTD, Zhengzhou, 450001, China

**Keywords:** Plant biology, Systems biology, Bioinformatics, Genetics, Transcriptomics, Sequencing, Variation, Parallel

## Abstract

As the development of sequencing technology, it is now possible to sequence individuals of each species. Although a number of different tools have been developed to detect individual variations, most of them cannot be run in parallel modes. To accelerate variation detection, PVCTools is introduced in this study. PVCTools splits the reference genome and alignment files into small pieces and runs them in parallel mode. Meanwhile, boundary noise is also considered in PVCTools. From the result of three different sets of test data, PVCTools performs much faster than most other current tools. At the same time, it keeps similar accuracy with other tools. PVCTools is free and open source software. The development of sequencing technology and growing sample numbers will make performance improvements such as PVCTools increasingly interesting.

## 1 Introduction

In the past few years, there have been many advances made in the high throughput sequencing technologies (Altmann et al., 2012), including Illumina, Applied Biosystems SOLiD System, 454 Life Sciences, Helicos HeliScope, and, most recently, PacBio and Nanopores. Due to these advances, the genomes of many species have been released. Hence, it is now possible to detect potential disease/trait-related variants. Sometimes, next generation sequencing (NGS) data is even used for diagnostic purposes. This is partially due to the development of sequencing technologies and the capacity of the various bioinformatics tools developed to analyze the growing numbers of sequencing datasets (Mielczarek and Szyda, 2016).

To detect genetic variants that mark individual variations as distinct from the reference genome, a number of tools have been developed, including GATK-HC (Genome Analysis Tool Kit Haplotype Caller) (McKenna et al., 2010), samtools mpileup (Li et al., 2009a), freebayes (Garrison and Marth, 2013), SOAPsnp (Li et al., 2009b) and sambamba (Tarasov et al., 2015). Although these tools have some advantages and disadvantages, they have been used to identify a large number of useful variations for disease diagnostics and crop breeding (Zhou et al., 2016).

As the price of sequencing drops, more individuals are being sequenced to compare population genetic differences. However, most of the current tools are single-thread or cannot be run on multiple nodes (Table 1). Although sambamba supports multiple threads (Tarasov et al., 2015), the jobs still cannot be assigned onto multiple nodes. This issue also exists in Freebayes-parallel. A new library of GATK (GenotypeGVCFs) has also been introduced to run on multiple threads, however, the final quality score of each variation only uses average values, which may be unacceptable. Spark-GATK (https://github.com/PAA-NCIC/Spark-GATK) supports multiple nodes, which is built on spark cluster and could not run on other popular clusters, such as LSF. Therefore, the speed of data analysis is still the bottleneck, especially for multiple samples of large genomes. Addressing the speed problem matters for both diagnostics and analyzing and sharing computation resources. Introducing new tools supporting multiple threads and nodes can accelerate the variation detecting process.

**Table 1:** Comparison of different variation detection tools.

Software	Multi-sample	Multi-thread	Multi-node
samtools	Yes	No	No
GATK	Yes	Yes	Yes
SOAPsnp	No	No	No
freebayes	Yes	Yes	No
sambamba	Yes	Yes	No

To accelerate variation detection, we developed PVCTools (Parallel Variation Calling Tools), a new software that fully utilizes the parallel processing resources of the server. PVCTools is written in C++, and all internal jobs can be assigned on different nodes and threads. In this way, PVCTools can run much faster than current variation detection tools.

## 2 Results

### 2.1 Datasets

To test the performance of PVCTools, three different datasets, from Arabidopsis (http://mtweb.cs.ucl.ac.uk/mus/www/19genomes/), Rice (http://iric.irri.org/resources/3000-genomes-project) and Human (http://www.internationalgenome.org/data/), with different genome sizes, were selected (Table 2).

**Table 2:** The information for three test datasets.

Species	Genome size	Number of samples	Sequencing depth
*Arabidopsis thaliana*	125 Mb	17	36.7X
*Oryza sativa*	420 Mb	20	10.0X
*Homo sapiens*	3.2 Gb	20	7.7X

### 2.2 Benchmark

We compared PVCTools with other popular tools (samtools, GATK, freebayes and sambamba) for performance and accuracy.

For performance, we compared PVCTools with other tools in speed and memory usage.

For accuracy, three criteria were defined and measured. If we define **m** as the variations detected by PVCTools, **n** is the variations detected by other tools. **l** is the intersection between **m** and **n**, and **p** is the number of different variations between **m** and **n**.

▪ Missing rate

$$\frac{the\;number\;of\;p}{the\;number\;of\;m}$$

▪ Difference rate

$$\frac{the\;number\;of\;variation\;in\;l,\;which\;score\;difference\;\geq 5\text{\%}}{the\;number\;of\;l}$$

▪ Correlation

The correlation of variation scores in l (score difference ≤5%).

### 2.3 The results of the Arabidopsis dataset

**Fig. 1:**
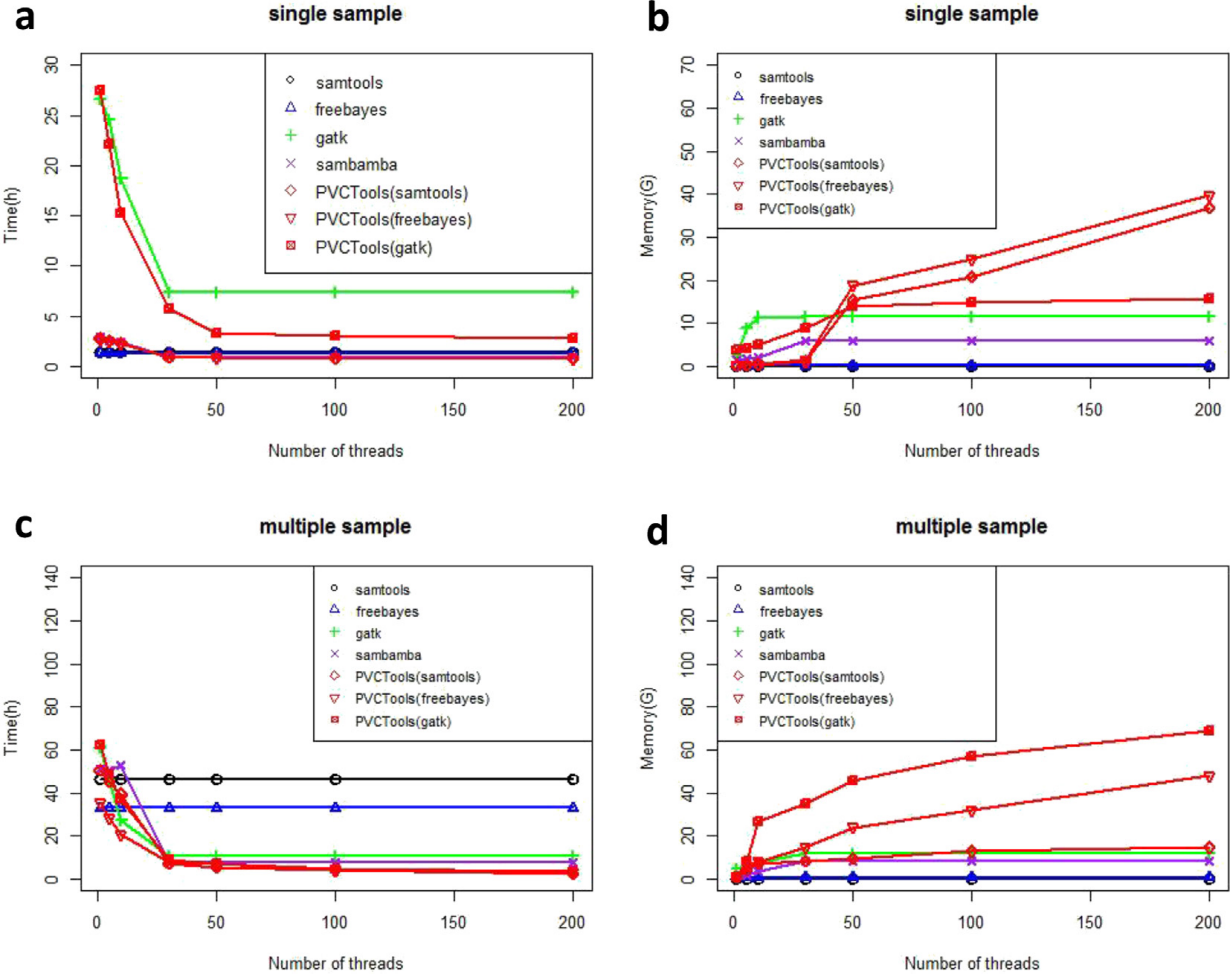
The result for the Arabidopsis test dataset. (a) Speed comparison between PVCTools and other tools using a single sample dataset. (b) Memory comparison between PVCTools and other tools using a single sample dataset. (c) Speed comparison between PVCTools and other tools using a multiple sample dataset. (d) Memory comparison between PVCTools and other tools using a multiple sample dataset. Note: X-axis represents the available computer resource for each tool.

For each test dataset, we compared PVCTools with other tools using single sample and multiple sample datasets. From the results for the Arabidopsis dataset (Fig. 1), we find that PVCTools has similar speeds to samtools, freebayes and sambamba, around 1–2 h. PVCTools is a little faster than GATK, especially using more than 30 threads (more than one nodes). However, for multiple sample analysis, PVCTools runs much faster than other tools, especially when using more CPUs on multiple nodes. For example, the time is reduced from around 50 h to around 10 h. Although the memory usage of PVCTools is much larger than other tools (around 40GB), the resource is not very limited for current servers.

**Fig. 2:**
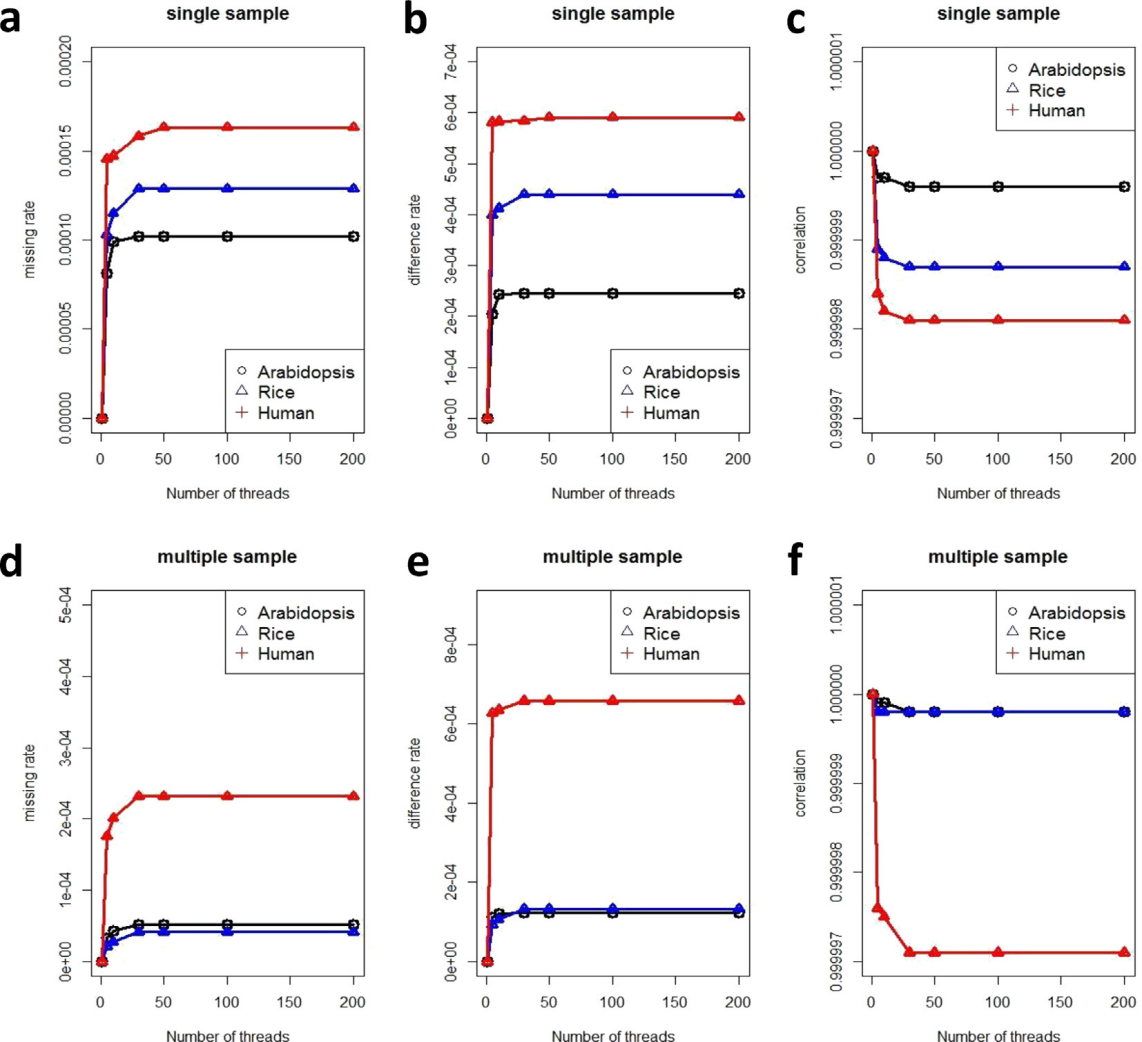
Accuracy comparison between PVCTools and samtools. (a) Missing rate comparison between PVCTools and samtools for a single sample. (b) Difference rate comparison between PVCTools and samtools for a single sample. (c) Correlation comparison between PVCTools and samtools for a single sample. (d) Missing rate comparison between PVCTools and samtools for multiple samples. (e) Difference rate comparison between PVCTools and samtools for multiple samples. (f) Correlation comparison between PVCTools and samtools for multiple samples. Note: X-axis represents the available computer resource for each tool.

**Fig. 3:**
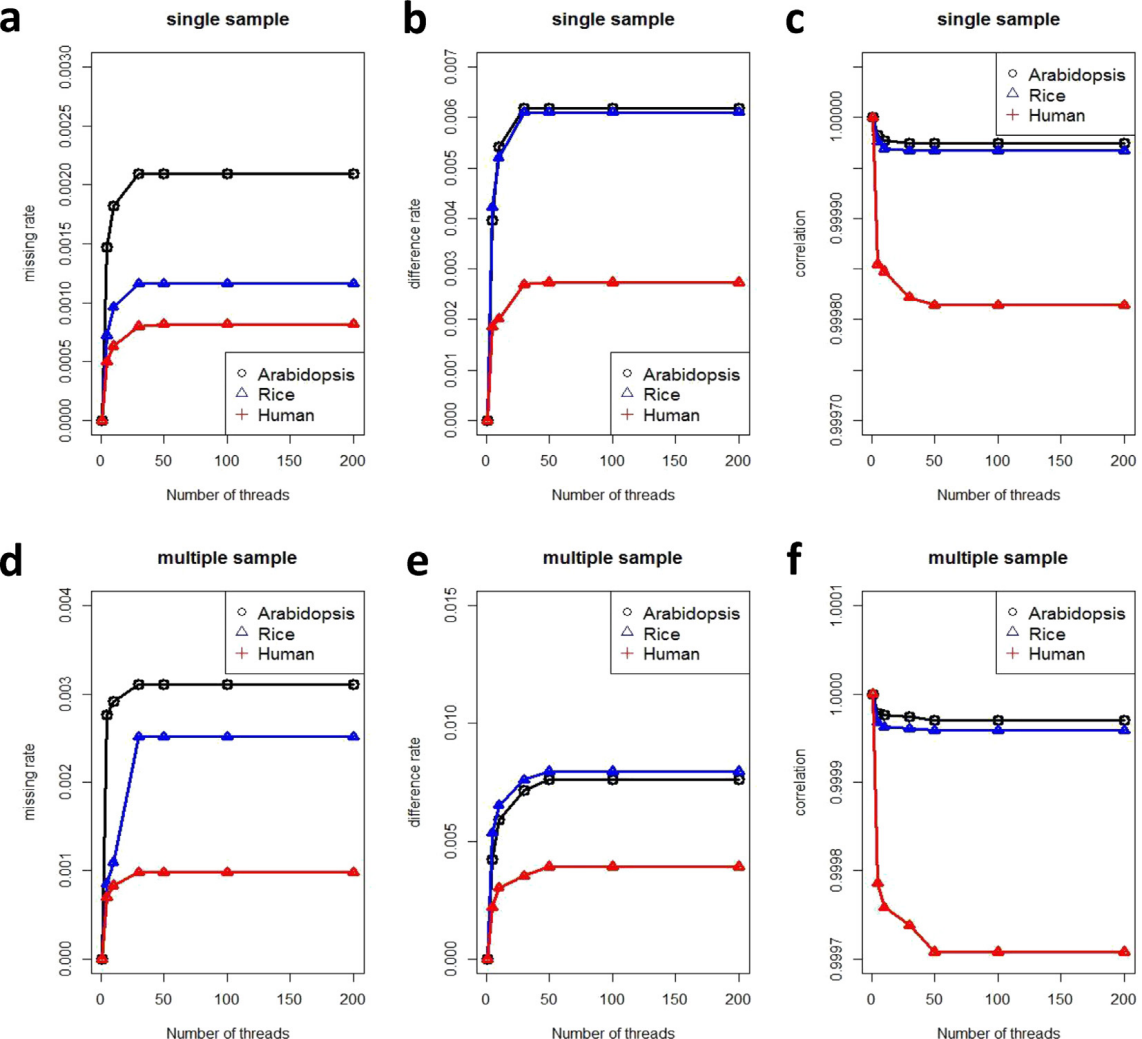
Accuracy comparison between PVCTools and GATK. (a) Missing rate comparison between PVCTools and GATK for a single sample. (b) Difference rate comparison between PVCTools and GATK for a single sample. (c) Correlation comparison between PVCTools and GATK for a single sample. (d) Missing rate comparison between PVCTools and GATK for multiple samples. (e) Difference rate comparison between PVCTools and GATK for multiple samples. (f) Correlation comparison between PVCTools and GATK for multiple samples. Note: The X-axis represents the available computer resource for each tool.

**Fig. 4:**
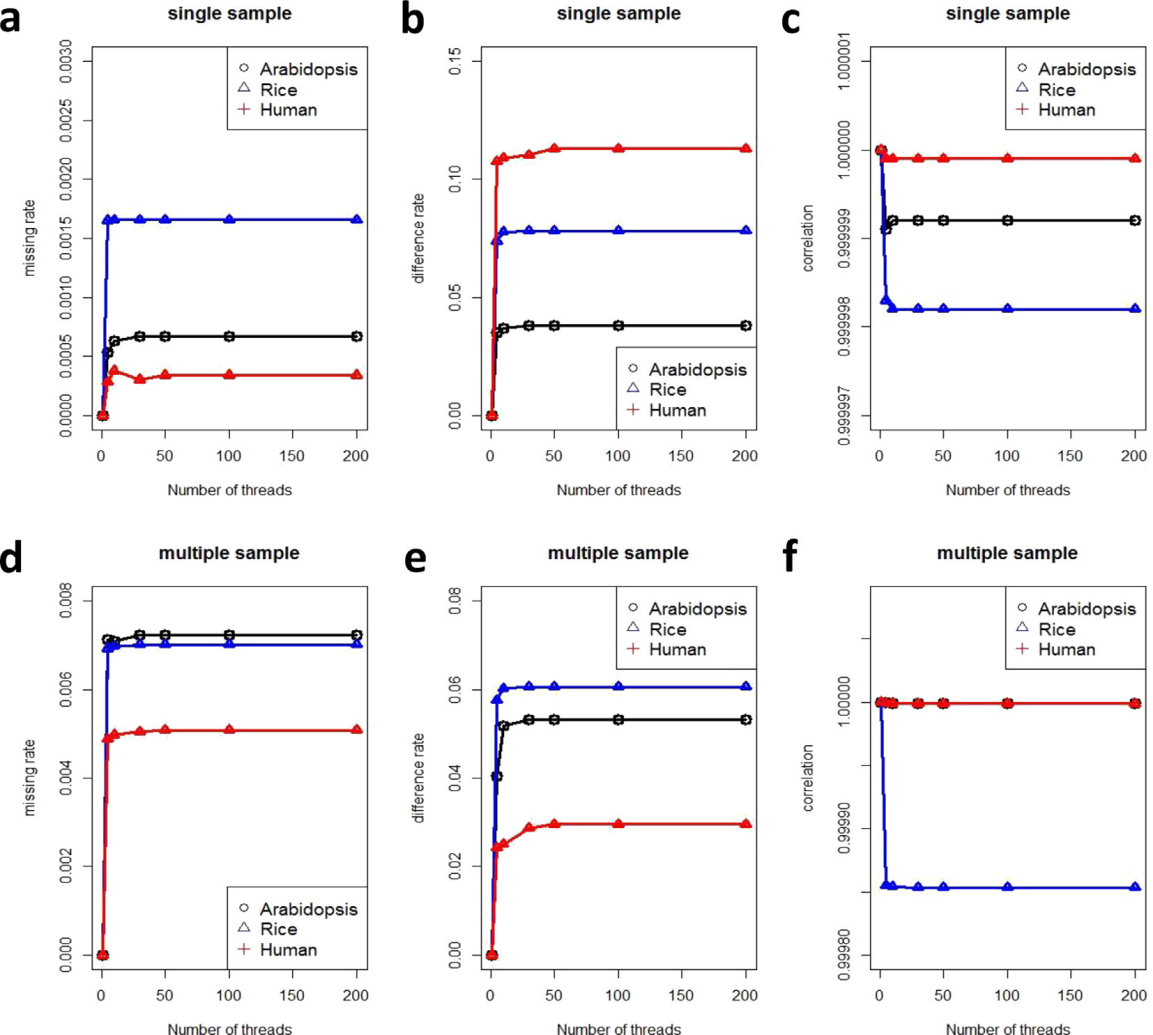
Accuracy comparison between PVCTools and freebayes. (a) Missing rate comparison between PVCTools and freebayes for a single sample. (b) Difference rate comparison between PVCTools and freebayes for a single sample. (c) Correlation comparison between PVCTools and freebayes for a single sample. (d) Missing rate comparison between PVCTools and freebayes for multiple samples. (e) Difference rate comparison between PVCTools and freebayes for multiple samples. (f) Correlation comparison between PVCTools and freebayes for multiple samples. Note: The X-axis represents the available computer resource for each tool.

Moreover, as shown in Fig. 2, compared to samtools, the difference rate for PVCTools is approximately 1/1000, the missing rate is approximately 1/10000, and the correlation for PVCTools is more than 0.99. Similar results are also found for GATK and freebayes (Figs. 3 and 4). In other words, PVCTools is faster than other tools while maintaining similar accuracy.

### 2.4 The results of the rice dataset

**Fig. 5:**
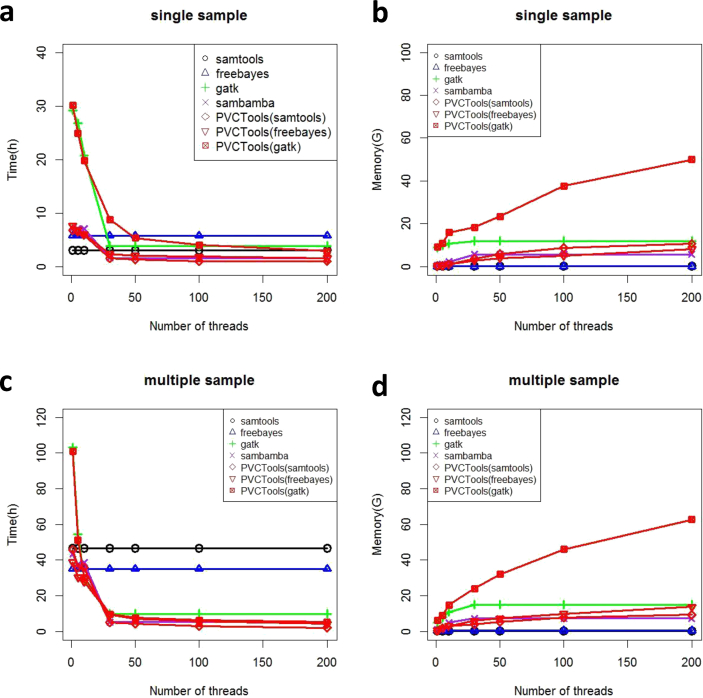
The result for the Rice test dataset. (a) Speed comparison between PVCTools and other tools using a single sample dataset. (b) Memory comparison between PVCTools and other tools using a single sample dataset. (c) Speed comparison between PVCTools and other tools using a multiple sample dataset. (d) Memory comparison between PVCTools and other tools using a multiple sample dataset. Note: X-axis represents the available computer resource for each tool.

Similar results can be found for the Rice dataset (Fig. 5). PVCTools has similar speed to samtools, freebayes and sambamba, around 3–5 h. PVCTools is a little faster than GATK, especially using more than 30 threads (more than one nodes). However, for multiple sample analysis, PVCTools also runs much faster than other tools (sometimes 3–4 times, from around 50 h to 6–7 h). Memory usage of PVCTools with the Rice dataset is also similar to that of the Arabidopsis dataset. For accuracy comparisons, we also reach similar conclusions (the difference rate is approximately 1/1000, the missing rate is approximately 1/10000, and the correlation is more than 0.99) (Fig. 2).

### 2.5 The results of the human dataset

**Fig. 6:**
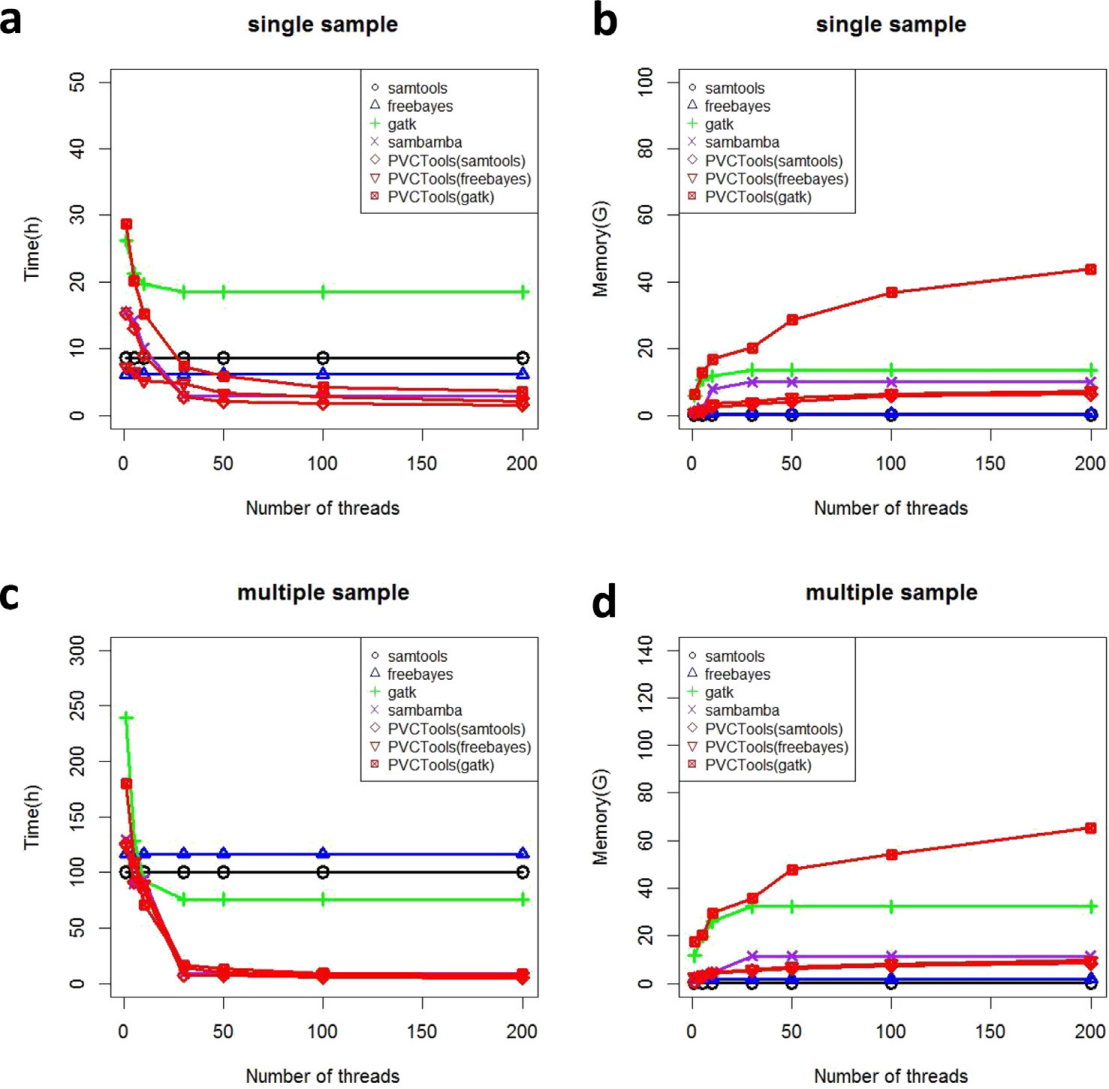
The result for the Human test dataset. (a) Speed comparison between PVCTools and other tools using a single sample dataset. (b) Memory comparison between PVCTools and other tools using a single sample dataset. (c) Speed comparison between PVCTools and other tools using a multiple samples dataset. (d) Memory comparison between PVCTools and other tools using a multiple samples dataset. Note: X-axis represents the available computer resource for each tool.

PVCTools has similar speeds to samtools, freebayes and sambamba and is a little faster than GATK on the human dataset (Fig. 6). For multiple sample analysis, PVCTools runs much faster than other tools (sometimes more than 5 times, from around 150 h–20 h). In other words, for sequencing datasets from large genomes, PVCTools has more advantages than other tools. Even for a large genome, such as the human genome, memory usage (around 80GB) is still acceptable for most servers. The most important characteristic is that PVCTools also maintains high accuracy (the difference rate is approximately 1/1000, the missing rate is approximately 1/10000, and the correlation is more than 0.99) (Fig. 2).

## 3 Conclusion

PVCTools is a parallel variation calling tool that tries to fully utilize computer resources. By splitting reference genome and alignment files, PVCTools achieves improvements in speed. The results on three different test datasets reveal that PVCTools not only is a faster variation calling process but also achieves a similar level of accuracy as other tools. The reduction of sequencing costs and the growing number of samples makes such performance improvements increasingly relevant.

## 4 Materials and methods

### 4.1 Implementation

PVCTools is a Linux-based pipeline implementing a fully integrative analysis workflow. Standard input and output formats, such as fasta/fastq, BAM and vcf output formats are used to facilitate users. The architecture of PVCTools contains four steps, consisting of the following:

▪ Split the reference genome into smaller pieces
▪ Split alignment files (BAM) into smaller pieces
▪ Run variation detection jobs in multiple nodes and threads
▪ Merge variation files and modify location of variation

**Fig. 7:**
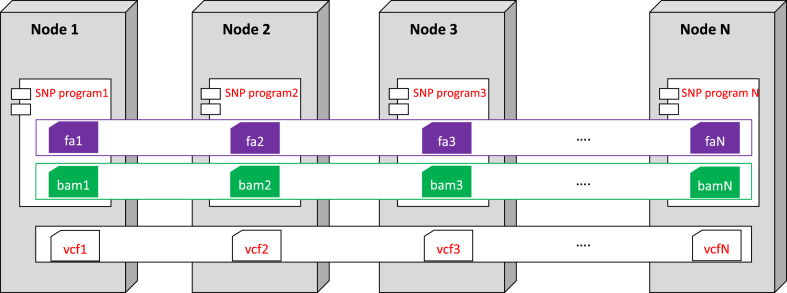
The pipeline of PVCTools.

Each step is described in detail below, and the workflow of the pipeline is visualized in Fig. 7. More details for usage are described at https://github.com/CNaibon/PVCTools.

#### 4.1.1 Step one: split the genome

PVCTools will split relative inputs into smaller pieces to accelerate data analysis. Therefore, the reference genome file is first split into smaller pieces. Users can split the reference genome according to the chromosomes. Moreover, each chromosome can be cut into much smaller pieces according to requirements. At the same time, the modified location for the splitting place is recorded for further analysis. To reduce noise in the boundary of splitting a genome, a region (longer than the sequencing reads) will be kept for each piece.

#### 4.1.2 Step two: split the alignment file

According to the split results from step one, the alignment file is further split. Finally, each splitting piece from the reference genome will have a corresponding alignment file from each sample. Meanwhile, the map location will be modified for the new relative locations in the reference genome, and the absolute location will also be recorded for further analysis.

#### 4.1.3 Step three: run the variation calling jobs

Based on the job mode in the operating system, batch variation calling jobs will be generated for different pieces from the reference genome. PVCTools supports samtools, GATK and freebayes to detect variations. These jobs will be parallel run in different threads and nodes according to the user's requirement. The corresponding result file (vcf) will be obtained for each job, and the relative location for each vcf file will be modified to an absolute location.

#### 4.1.4 Step four: merge the variation results

The corresponding vcf result files will be merged into the final result file for users.

According to the above algorithm, there are nine modules in PVCTools, including GetVCF, SplitFA, SegmentFA, SplitBAM, SegmentBAM, Submit, SmallFA and Environment.

▪ GetVCF: This module executes all the modules in PVCTools, and outputs the final variation vcf file to users.
▪ SplitFA: This module splits reference genome file by chromosome.
▪ SegmentFA: This module splits longer chromosome into smaller pieces.
▪ SplitBAM: This module splits alignment file by chromosome.
▪ SegmentBAM: This module splits alignment file related to longer chromosome into smaller pieces.
▪ Submit: This module submits batch jobs and assigns them onto multiple nodes/threads.
▪ SmallFA: This module executes batch jobs related to smaller chromosome.
▪ Environment: This module changes environment parameters.

Users can run the entire set of modules with one command and can run each individual module according to their requirements.

### 4.2 Availability and requirements

**Project name**: PVCTools

**Project home page**: https://github.com/CNaibon/PVCTools

**Operating system**: Linux (20 nodes)

Cluster: LSF

CPU: 12 cores (4 Intel Gold-6126)

RAM: 512G

**Programming language**: C++

**Other requirements**: samtools, GATK, bamUtil, freebayes

**License**: GNU GPLv2

**Any restrictions to use by non-academics**: None

## Declarations

### Author contribution statement

Jingjing Jin: Conceived and designed the experiments; Performed the experiments; Wrote the paper.

Jiajun Liu: Conceived and designed the experiments; Performed the experiments.

Yelin Yin, Peng Lu, Jianfeng Zhang: Analyzed and interpreted the data.

Zefeng Li: Analyzed and interpreted the data; Contributed reagents, materials, analysis tools or data.

Yalong Xu: Contributed reagents, materials, analysis tools or data.

Peijian Cao, Dasha Hu: Conceived and designed the experiments; Wrote the paper.

### Funding statement

This work was supported by the Projects of Parallel Variation Calling Tools (902015CA0250), ENCODE of Tobacco Genome (110201601033 (JY-07)) and the Young Elite Scientists Sponsorship Program by CAST (2016QNRC001).

### Competing interest statement

The authors declare no conflict of interest.

### Additional information

No additional information is available for this paper.
